# Scoping review of mental health-related policies issued in the context of the COVID-19 pandemic in Peru

**DOI:** 10.1371/journal.pmen.0000459

**Published:** 2026-04-27

**Authors:** Victoria Cavero, Ana L. Vilela-Estrada, Rubí Paredes-Angeles, Noelia Cusihuaman-Lope, David Villarreal-Zegarra, Francisco Diez-Canseco

**Affiliations:** 1 CRONICAS Center of Excellence in Chronic Diseases, Universidad Peruana Cayetano Heredia, Lima, Peru; 2 Instituto Peruano de Orientación Psicológica, Lima, Peru; PLOS: Public Library of Science, UNITED KINGDOM OF GREAT BRITAIN AND NORTHERN IRELAND

## Abstract

This study aims to identify, describe and analyze the policies enacted by the Peruvian health authorities to address the population mental health needs and adequate health services in the context of the COVID-19 pandemic. We conducted an scoping review of mental health-related policies published between 6 March 2020 (first case of COVID-19 in Peru) and 31 May 2023. The official websites of the Peruvian Government, the Social Security Health System (EsSalud), and the LILACS scientific database were used. Data was analyzed using content and thematic analysis. Eighty-two documents were included. Policies contents were divided into three main categories. First, the governance of mental health care, which portrays the principles guiding policies and those seeking to strengthen mental health services by increasing the allocation of budget, use technology to provide remote care, enhance infrastructure, equipment and materials, hiring and training staff, and assure biosafety measures. Second, service delivery of mental health care, including policies aimed to detect and provide specialized mental health care to different groups of the population. Third, some policies focused on community engagement, promotion, and prevention in mental health, such as reducing misinformation, promoting self-care, community participation, and working with local organizations. We found that Peru included most of the international recommendations, but some important weaknesses were found, such as the lack of indicators to assess their completion or procedures on how to adapt these policies to routine practices. Peru designed several policies to attend to the mental health of its population during the COVID-19 pandemic, specifically on the principles for the provision of mental health care, the strengthening of these services, detection of mental disorders, provision of mental health care, and care at the community level. This study will inform other countries on their design of mental health policies, especially those with a community model-of-care.

## Introduction

Governments around the world proposed a series of measures to mitigate the exponential expansion of the COVID-19 pandemic, which took millions of lives, and to avoid the overburden of their health systems, such as mandatory social isolation (quarantine), social distancing, restriction of movement hours (curfews), use of telemedicine instead of in-person care, suspension of transport, and closure of borders [1]. However, these measures affected people's routines by suspending their work and academic activities, loss of income, the closure of commercial stores, entertainment and sports centers [2], and increased the overburden of health workers, especially those treating cases of COVID-19 [2].This context laid the ground for a major negative impact on people's mental health, increasing the prevalence of clinical and subclinical disorders, such as acute stress, depression, anxiety, post-traumatic stress disorder (PTSD), and other mental health symptoms [3].

To guide governments on how to attend the mental health of their populations, several international guidelines were published by global agencies and mental health experts. Here we present some of them. First, the Inter-Agency Standing Committee (IASC) Reference Group on Mental Health and Psychosocial Support (MHPSS) in Emergency Settings published a briefing note addressing the MHPSS in the context of the COVID-19 outbreak [4]. It addresses the psychological and social effects of the pandemic, such as stress, fear, anxiety, and stigma, in the general population and vulnerable groups (older adults, children, people with disabilities, and front-line workers). The document proposes interventions at multiple levels, from integrating social and cultural considerations into basic services to providing specialized services for people with severe conditions. It also highlights key approaches such as human rights, equality, participatory, and integrated support systems.

Second, the World Health Organization (WHO) conducted a survey to 130 countries in 2020 about the impact of the pandemic on mental health services and how countries handled these issues [5]. This survey collected pivotal information on how governments tackled the mental health of their populations during the COVID-19 pandemic, including multi-sectorial coordination, continuity of services, strategies to overcome services disruption, and surveillance mechanisms.

Third, a group of global mental health experts reflected on the impact of the COVID-19 pandemic in the mental health of people living in low- and middle-income countries (LMICs) and how governments could improve their responses to the emergency and the mental health policies in their countries [6]. Authors outlined a series of interventions for detection and care of mental health conditions as well as infectious disease control efforts integrating mental health principles. Finally, they provided some recommendations to build back better, such as integrating mental health services into universal health coverage, improving access and coverage to psychosocial interventions, eliminating coercion in mental health care, among others.

Fourth, a group of early career psychiatrists proposed a conceptual framework of mental health interventions for preparation and action during the COVID-19 pandemic [7]. They proposed five main categories: 1) Preparation and coordination, including surveillance, training, and specific care; 2) Monitoring and assessment, prioritizing populations at risk and the use of technologies; 3) Reducing mental distress due to misinformation about COVID-19; 4) Sustainability of mental health services, through funding, policy, and coordination; and 5) Communication through mass media and digital support groups.

Finally, another article published by global mental health experts explored the potential impact of the COVID-19 pandemic on populations mental health, suggesting some actions to improve their care [8]. Authors provided a series of guidelines and interventions for specific populations: General population, people with mental health conditions, people delivering essential services (i.e., health workers), and people who suffered the COVID-19 illness.

To reduce the impact of the COVID-19 pandemic on their populations mental health, several countries rapidly formulated and implemented mental health policies in this regard. However, due to the disruptive nature of the emergency, several were not properly analyzed nor improved. For instance, Chile [9], China [10], Kenya [11] or Rwanda [12], tried to assess their health responses during the pandemic and recommended that as the pandemic context progressed, it was necessary to adjust and evaluate their public health policies.

This paper outlines the experience of Peru, one of the most affected countries by the pandemic, which reached more than 200,000 deaths until 2022 and had a severe impact on the mental health of its population [13], issuing mental health-related policies during the COVID-19 pandemic [14]. Only in May 2020, 30% of the Peruvian adult population reported having experienced any depressive symptoms [15], and 33% of children and adolescents experienced mental health problems associated with the pandemic [16]. Similar to other countries, the Peruvian government issued several policies to strengthen its public health system [17] to address the mental health impact of the COVID-19 pandemic in its population [18].

This study aimed to retrospectively identify and describe thematic priorities of the mental health-related policies designed by the Peruvian health authorities to address the population's mental health needs and adequate the health services in the context of the COVID-19 pandemic, as well as assess policies completeness relative to the international benchmarks detailed above [4–8].

## Methods

### Study setting

In Peru, the Ministry of Health (MoH) oversights the healthcare sector in the country and thereby its policies must be implemented nationwide. Yet, the health system is very fragmented in terms of funding sources, insurance schemes, and health services [18], so that different health systems can adapt the MoH policies and propose their own. There are four main health systems in Peru: 1) the MoH, which allocate its budget through Regional governments, hospitals, and local governments, and has the *Seguro Integral de Salud* (SIS), which is funded by taxes and is directed to those living in poverty; 2) Social Security Health System (EsSalud), which is funded by payroll discounts of formal workers and is administered by the Ministry of Labor; 3) the Army and Police, which are funded by the Ministry of Defense and is directed to the Army, Police and their relatives; and finally, 4) several private insurances [19]. This study prioritized the health policies issued by the MoH and EsSalud because they cover together the 90% of the population [18].

The Regional Health Directorates are decentralized offices of the MoH in the different regions of the country. They are responsible for the Health Networks Directorates, in charge of health services at different levels, and specialized hospitals [19]. Since Regional Health Directorates and specialized Hospitals can propose their own policies based on the specific needs of their populations, we also included their policies.

Finally, Community Mental Health Centers (CMHC) were created as part of the Mental Health Reform in Peru to provide mental health care to people with severe mental disorders [20]. CMHCs are a key component of the community-based model of care that seeks to decentralize the provision of mental health care in the country. In the last years, Peru has increased the number of CMHCs nationwide, from 29 CMHCs in 6 regions in 2017 to 248 CMHCs in all regions by 2023 [20,21]. These centers have basic teams composed of medical doctors, psychiatrists, psychologists, nurses, and social workers, in addition to occupational and language therapists.

### Study design and protocol register

We conducted a scoping review of policies at the national- and regional-level published between 6 March 2020 and 31 May 2023 in Peru, which was the period in which the government issued policies to reduce the impact of the pandemic and adequate its health services to the new context. The Preferred Reporting Items for Systematic reviews and Meta-analysis extension for Scoping Reviews (PRISMA-ScR) Checklist [22,23] was used (S1 File). The protocol of this study was published in the Open Science Framework (https://osf.io/tf9h6/).

### Data sources

Documents included were different types of policies, such as directives (mandatory policies), plans (broader management tools), guidelines (detailed non-mandatory documents), or related documents that addressed the government response to the mental health impact of the pandemic in Peru (gray literature). Documents were selected from one scientific database (LILACS), the official website of the Peruvian government (https://www.gob.pe/)- which gives access to the MoH, Regional Health Directorates, and hospitals policies, and The Social Security Health System (EsSalud in Spanish) (http://www.essalud.gob.pe/ietsi/index.html)- the second main health system in Peru that provides care for all formally employed workers. All documents were written in Spanish, the country official language.

### Search strategies

The time-frame to select the documents was from 6 March 2020 (date of the first COVID-19 reported case in Peru) to 31 May 2023 (month in which the WHO declared the end of the pandemic as a public health emergency). During this period, the main policies on the response of the Peruvian government were enacted. The search strategy included a combination of the terms “psychosocial”, “mental health” and “COVID-19”, which was tailored to each database or website used, as shown in S2 File. Four members of the research team were involved in this process.

### Eligibility criteria

Policy documents were included if they met the following criteria:

**Inclusion criteria:**

- Published between 6 March 2020 and 31 May 2023.
- Issued by a public health institution (i.e., MoH, public hospital, etc.).
- Stated the context of the COVID-19 pandemic.
- Provided any mention of mental health or psychosocial care.
- Targeted to the Peruvian population as a whole or specific Peruvian sub-populations.

**Exclusion criteria:**

- Press releases that mention a policy but do not contain or redirect to any document fulfilling inclusion criteria.
- Documents related to settings different from Peru.
- Duplications.

### Selection of sources of evidence

All identified documents were collated and uploaded into Zotero. Duplicates were removed. Then, titles and abstracts were screened by two research team members. In the second stage, the full-text of selected records were reviewed in detail against the inclusion criteria by the same two team members. Disagreements between reviewers were solved through discussion.

### Data extraction

All documents identified in the selected database or websites were screened by two independent reviewers, who first reviewed documents by title and aim, and then screened those selected in full text. Disagreements between reviewers were discussed upon reaching an agreement. Information was extracted in a matrix, including title of the policy, institution/author, document official ID, if applicable (i.e., #999–2021-MoH), date of publication, link, and summary of the main contents.

### Data synthesis and analysis

Data were analyzed and synthesized using a code-book developed by the research team based on the contents of international guidelines addressing the provision of mental health services during the COVID-19 pandemic [4–8]. We followed three steps. First, basic information (columns) from each document (rows) was extracted in a matrix in Excel (e.g., title, author, publication date). Second, using content analysis [24] all included documents were summarized into the code-book built in the same matrix in Excel. This code-book was divided in four different sections, according to four target populations identified in the international guidelines [4,8]: Policies for the general population, policies for people with mental disorders, policies for people with COVID-19, and policies for people delivering essential services (e.g., health workers, police, salespeople). Each section had a series of codes that can be seen in S3 File. In each cell, the research team included a summary of the policy content, quotations from the policy text and page numbers.

This process was conducted by two members (VC- AVE) of the research team with extensive experience in mental health and well-known of the community-based mental health system in Peru. Four members of the research (VC- AVE-RPA-DVZ) team built the code-book and then met to standardize the coding process. First, they coded one policy document separately and contrasted their coding in group to assess similarities and the rationale used. They repeated this process with two more documents but only two members (VC- AVE) reached an inter-coder agreement of 95% (the others, with less experience in qualitative research, reached less than 60%), so only the more experienced team members continued coding the full dataset. Along with the coding process, doubts were discussed together and disagreements were solved with the other two members of the team.

Finally, using thematic analysis [25] data from each section (i.e., policies for the whole population, policies for people with mental disorders) was gathered together and summarized, identifying the main themes covered by the policies. The results section was organized based on this categorization of themes. Importantly, this study focuses only on the contents of the policies and what should be happening in the public health services in Peru, not in their implementation, since this is not specified in the policy documents.

### Ethics approval and consent to participate

The study was a scoping review of open access documents, which did not require ethical approval nor the consent of any human person to participate.

## Results

### Selection of sources of evidence

**Fig 1:**
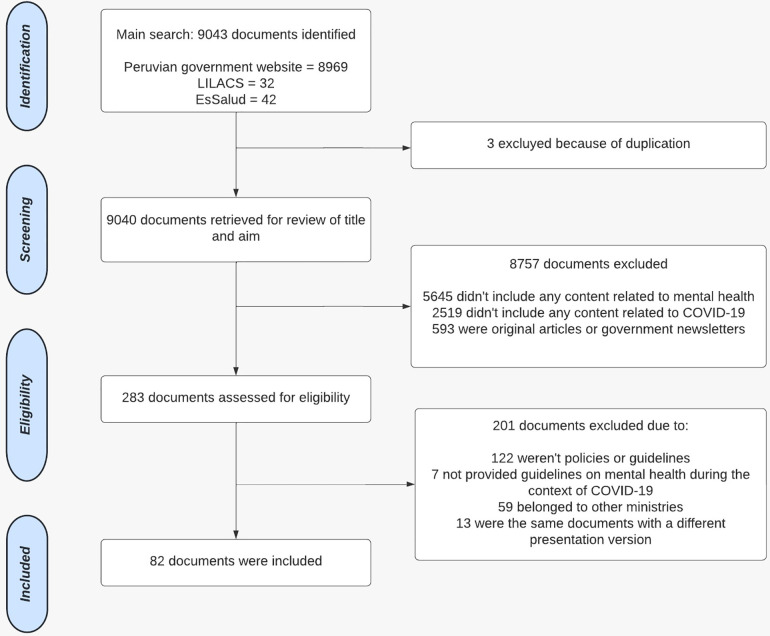
The flow diagram of policy identification and search.

The search yielded a total of 9043 policy documents. First, 3 duplicates were removed and 9040 remained. Then, 8757 policy documents which did not meet the eligibility criteria were eliminated during the revision of title and aim, leaving 283 documents for further scrutiny. After screening full-texts, 201 documents were excluded and 82 were finally included for analysis (see Fig 1).

### Characteristics of sources of evidence

Of the 82 documents included in the study, 51 were issued by the MoH, 12 by EsSalud, 11 by Regional Health Directorates, and 8 by public hospitals. Identified policies from the MoH were usually broad to ease their implementation in diverse country settings, considering their national scope, and sometimes included different formats of the same document (i.e., shorter, more colorful, with graphics, etc.) to ease their reading by health workers. The MoH proposed policies aimed at providing mental health care for the general population, emphasizing measures related to strengthening the mental health of communities and the facilities physical and digital infrastructure; as well as assuring the availability of appropriate materials for the provision of mental health services (i.e., personal protective equipment, printed or digital screening tools). MoH’s policies usually lacked specific procedures to ease the incorporation of their guidelines into routine practices. For example, some policies suggested migrating from in-person to virtual care, but there were no details on how to do it, nor what tools or platforms should be used.

EsSalud’s policies, on the other hand, were all developed by the “Institute for Technological Evaluation and Research in Health (IETSI)” and were mainly linked to the detection of mental disorders and the provision of mental health care among people with COVID-19, their families, and workers who provided essential services during the pandemic. These policies were usually supported by rapid systematic reviews, providing specific details on their possible outcomes, screening tools, and the evidence behind their recommendations.

Finally, policies issued by the Regional Health Directorates and the directorates of public hospitals were mainly focused on adapting the MoH policies contents that matched their specific contexts. For instance, general hospitals included guidelines for hospitalized patients with COVID-19 or mental health treatment to their health workers.

The MoH and EsSalud developed 25 documents (S4 File) with exclusive mental health content; whereas the other 57 documents (See S5 File) were health policies that included some contents on mental health. One of the main documents was the National Mental Health Plan adapted to the needs and the context of the pandemic, to promote the mental health of the general population, and to provide support to people with mental disorders [26]. This comprehensive policy was taken as a reference for health centers nationwide to adapt their provision of mental health care [27–29]. In general, most of the 82 enacted policies focused on the detection and treatment of mental disorders, especially for patients with COVID-19 and their families. In contrast, there were few guidelines about mental health promotion and prevention during the pandemic.

### Specific contents of the mental health-related policies

#### Governance of mental health care.

##### Principles for the provision of mental health care:

The reviewed policies included some approaches to support the provision of mental health services during the pandemic, such as human rights, gender, disability, and interculturality. These policies prioritized specific vulnerable populations, such as children, adolescents, women, older adults, LGTB community, and ethnic minorities [26,27,29–31]. Many documents emphasized the importance of providing treatment without discrimination to COVID-19 patients and their families [29,31–33] and sought to ensure the continuity of psychological and pharmacological treatment for patients with pre-existing mental health problems and those diagnosed during the pandemic [26,29,30,34,35]. In addition, all health centers with hospitalization services had to approve a psychosocial and communication support plan [36] and identify prioritized populations, which included people with mental disorders [26]. Moreover, all health centers should include mental health care in their plans and intervention strategies against the COVID-19 pandemic [31]. For psychiatric patients, some policies proposed to increase efforts for their deinstitutionalization, prohibit the use of coercive measures, use informed consent in interventions, and promote their integration into the community [31].

##### Strengthening mental health services:

Some policies were directed to the enhancement of mental health services, including an array of conditions: budget, use of technology to provide remote care, infrastructure, equipment and materials, human resources, and biosafety measures. In terms of financial resources, the Peruvian government annually allocates a budget for mental health. During 2020, the budget for mental health increased to cope with the second wave of the pandemic [37]. For 2021, the budget tripled compared to 2020 [26,38], passing from ~USD 30.5 millions in 2020 to ~USD almost 84 millions in 2021 [31,39]. In 2023, the MoH continued to prioritize mental health by including it in its annual operational plan, which allocated 1% of the institutional budget specifically to the prevention, early detection, and comprehensive care of mental health problems, with indicators to monitor progress, such as the proportion of people diagnosed and treated for affective disorders, anxiety, and the number of cases managed at the CHMCs [40].

Regarding the use of technology, the MoH promoted the use of telemedicine [41–47].National helplines were established for providing remote mental health services for promotion, prevention, diagnosis, recovery and rehabilitation [30,31,43,48], and information on COVID-19 and preventive measures [43].

For infrastructure, some policies indicated that health centers should have adequate infrastructure, which included relaxation rooms, beds for staff to rest, toilets, and spaces for physical activity [49]. Moreover, they had to adapt their clinics for differentiated care in two care circuits: COVID-19 and non-COVID-19 care to avoid contagion [43,45,50].

Regarding materials, some policies recommended the provision of working equipment and tools (e.g., personal protective equipment, telephone lines, cell phones, computers) and screening tools for mental health services at primary and secondary care level [28,31]. Additionally, the MoH received donations of COVID-19 rapid diagnostic kits directed to mental health services [51].

Regarding human resources, health centers should hire new staff to strengthen the provision of care in their mental health services, such as psychiatrists, psychologists, nurses, social workers, etc. [43,52]. Additionally, health centers formed new teams, called “non-COVID-19 teams”, which carried out their activities in-person, through home visits, or remotely [43]. Psychiatric hospitals received funding to hire and maintain their health personnel [53–55].

Additionally, some policies stated that all the health personnel had to receive training on psychological first aids [26,29,43], grief management, identification of mental health problems [31], telemedicine [26], use of technology [56,57], and identification and management of COVID-19 cases [57]. Likewise, those working in emergency services should have received training to treat psychiatric emergencies, recognize symptoms of COVID-19, and use technology [26,29,44,57–59]. Social actors (e.g., volunteers and community leaders) were also supposed to receive training on the access to psychosocial care and healthy practices for the prevention, containment, and mitigation of COVID-19 [58,59].

In terms of biosafety, health centers were supposed to guarantee adequate infrastructure and environments [31,36], by reducing the agglomeration of people, promoting social distancing [43,50,56], providing personal protective equipment to health, administrative and logistics workers at health centers [31,60], and assessing their patients before admission to health facilities (e.g., hand hygiene, use of a mask, use of face shield) [50,61].

#### Service delivery of mental health care.

##### Detection of mental health problems:

According to the analyzed policies, all public and private health centers were responsible for conducting mental health screenings and clinical interviews to identify risk factors, assess family support, and identify available community networks for people “affected by the COVID-19 pandemic” [30,56]. By affectation, the policies meant the mental health discomfort that the general population faced due to the pandemic and the government containment measures, as well as the mental health impact to those isolated at home or treated in hospitalization and emergency services due to COVID-19 [26,31,58]. The MoH prioritized some other populations to detect potential mental disorders, such as health personnel [26], children, adolescents, pregnant women [26,28–31,58,62,63], older adults [26,29,30], people with disabilities [26,29,31,56], and people who lost a beloved one due to COVID-19 [26].

Some policies also indicated that managers from all sectors should permanently assess the ergonomic, psychosocial and other health needs of their workers [64]. For health workers specifically, it was indicated that they should be screened to identify discomforts through observation and screening tools (e.g., Self Reporting Questionnaire (SRQ) for depression and anxiety, the Maslach Burnout Inventory for burnout, etc.) in group or individual activities [49,62].

#### Provision of mental health care tailored to targeted populations.

Most of the policies published by the MoH were related to guidelines for adapting mental health services through telemedicine, using actions such as tele-counseling, telemonitoring, tele-consultation, electronic prescription and dispensing of medicines [26]. Thus, some policies proposed to establish mechanisms for care considering that patients could decide the modality of care, such as video calls, and telephone calls [27,32,43,56,65]. It was identified that interventions should address grief, post-traumatic stress, suicidal behavior, anxiety, depression, alcohol abuse, psychosis, neurodevelopmental and emotional disorders [26], sexual and reproductive health and nutrition counselling for adolescents [56], violence, and sexual abuse [26,66]. This should be done in coordination with the emergency centers for women, which are specialized public institutions that provide psychological counselling, social support, and legal guidance to women victims of violence [30], police stations, the judiciary, educational institutions, youth centers, and shelters [46,56] depending on each case.

Another approach for people isolated at home was providing positive coping strategies, self-care guidelines [67], and a therapeutic intervention plan involving relaxation activities, provision of basic needs, rest, and adequate food to protect their mental health [31].

Specifically, policies established **that persons with severe mental disorders and/or psychosocial disabilities** under pharmacological or psychotherapeutic treatment should be prioritized for the continuation of their care [26,31]. In this regard, CMHCs should ensure access to psychotropic drugs through timely prescription and pharmacy access facilities to reduce the mobilization of patients in the context of a pandemic [29,31]. Therefore, they proposed that a) medication dispensing can be done with a physical medical prescription or with an electronic prescription in an outpatient clinic [43]; b) dispensing can be done in person at the health center to the patient or an authorized family member [14,27]; c) health personnel deliver the medication to the patient#39;s home or through grassroots organizations to reduce the number of patients attending the health services, and d) home delivery of medication [14,29,43]. In addition, the “Vaccination protocol for persons with severe mental disorders” was created, prioritizing vaccination for people with diagnoses of schizophrenia, psychotic disorders, bipolarity and neurodevelopmental disorders: autism spectrum disorder or intellectual disability [68].

**For people hospitalized for mental disorders**, it was essential to establish protective measures, such as restricting visits to health facilities, raising awareness about handwashing, limiting group meetings, and control measures for suspected, probable or confirmed cases of COVID-19 [61]. Likewise, in case of relapses or emergencies, persons with severe mental disorders and/or psychosocial disability should be cared for without any discrimination, guaranteeing biosecurity measures; emergency care cannot be denied to a person even in the condition of a probable or confirmed case of COVID-19 infection [31].

On the other hand, policies related to caring for **patients with COVID-19 and their families** were published since the guidelines consider family members as an essential element in the treatment of patients with COVID-19, highlighting the importance of keeping them informed about the health status of their relatives and attending to the impact that this generated [32,69,70].

The policies of the MoH indicated the creation and implementation of psychosocial support units for patients and their families [70], which consist of mental health teams in CMHCs and hospitals, led by a psychologist and supported by a psychiatrist, a nurse and a social worker; identify psychosocial risks, provide self-care guidance, psychosocial support, interventions, and 24-hour follow-up [31,37,70] to patients with COVID-19 (in home isolation and hospitalization), their families, and workers [26,39,62,67,71]. These activities can be face-to-face or remote, depending on the severity level of the patient and the dynamics of the health center.

1) **Mild cases of COVID-19 and with suspected infection isolating at home**: It was established as a policy to include an assessment of emotional symptoms by tele-guidance and tele-education to the patient [29,31,43,46,72,73]. Likewise, it was proposed to carry out a follow-up that includes a mental health care registry that explores and identifies emotional distress, possible mental disorders, and psychosocial problems [43]. This follow-up will also involve providing recommendations for mental health care [74], psychological first aid, positive coping strategies, self-care guidelines and a therapeutic intervention plan that includes relaxation activities, provision of basic needs, rest and adequate nutrition to protect their mental health [31]. In addition, the staff had to guarantee confidentiality to avoid stigmatizing patients and family members [29], and assign a mental health specialist for severe cases [72].
2) **Hospitalized COVID-19 cases:** For moderate cases, in hospitalization services, the team responsible for care had to provide psychological first aid to people treated for COVID-19 and their families [26,31,32,69]. In severe cases, when the patient is in the intensive care unit, it is proposed to guarantee pharmacological and non-pharmacological treatment, the latter aiming to reduce the number of people around the patient, providing spiritual support (based on religion) and facilitating video calls with their relatives [69]. During these calls, the health team must ensure psychological first aid during reports on the patient's health status and provide humane, warm treatment, active listening and validation of the family's expression of emotions [69].
3) **Family support:** If mental health problems or negative emotions were identified in the family group, the mental health team of the hospital or the CMHCs should provide psychosocial support [61] and carry out a timely intervention [33,70,75,76] through telemedicine to the families [31] to accompany them in the phases of mourning, achieve acceptance and emotional adaptation to continue with their life projects [29,32,43,70,77]. Finally, in the case of patients who died of COVID-19, CMHC must provide psychological support to families in the stages of mourning and symbolic burials and promote their adaptation to continue with their life projects [78].
4) **Patients with COVID-19 sequelae**: COVID-19 can cause psychiatric sequelae such as cognitive impairments, anxiety, depression, sleep disorders, and PTSD. Mental health assessments should prioritize these symptoms, especially in moderate and severe cases. Rehabilitation requires multidisciplinary teams, including medical specialists and mental health professionals, tailoring care to the patients condition. Health facilities must ensure teams from various specialties support recovery and reintegration. Comprehensive evaluations should start seven weeks post-discharge to detect ongoing or new symptoms. Mental health interventions vary by severity, from psychological therapies and telemonitoring for mild cases to psychiatric referral for severe symptoms. Standardized tools for delirium, quality of life, anxiety, and depression are recommended [79,80].

Regarding **health workers,** specific policies were identified that contemplated the creation of psychosocial support teams specialized in their care to provide different types of interventions, such as:

1) Individualized intervention plans include psychological first aid, psychosocial support, identification of biopsychosocial needs [39,49], relaxation activities, rest and healthy eating, socialization spaces and brief psychotherapeutic interventions [32]. Priority was given to acute stress, adjustment disorders, anxiety disorders, depressive symptoms, violence and burnout syndrome.
2) Group activities of emotional regulation and self-care; for which it is suggested to elaborate or use information sheets related to the prevention of stigmatization and social exclusion, provision of coping strategies, psychological first aid, burnout syndrome [48,60,81], active breaks, relaxation techniques and positive coping, and promote companionship, through graphic and group campaigns [14,27,32,44,49,59,71].
3) Follow-up of identified cases [45,57,62] and referral to CMHCs [49].
4) Coordination in case of psychiatric emergencies or home admission [39,49].

In 2022, the MoH included in their Internal Communication Plan a mental health campaign to sensitize their staff, disseminate existing mental healthcare channels, and provide emotional support [82]. Finally, EsSalud proposed mental health guidelines for workers return to work in all sectors [81], as well as indications of care and self-care at work for the mental health of health personnel [73].

Some other policies recommended prioritizing patients with cancer and pregnant women with suspected COVID-19 to access hospitalization for a multidisciplinary evaluation and management, including mental health care [28,76]. Other policies focused on women victims of violence [30,31,83], and the MoH adapted some general guidelines to the needs of this population, such as the protocol for joint action between health centers and emergency centers for women [30]. Finally, other policies prioritized the prevention of suicide for those at higher risk, specifically adolescents, youth, and COVID-19 survivors [84].

### Community engagement, promotion, and prevention in mental health

Some policies proposed disseminating information for the prevention of COVID-19 [29,31,56,61,73,85], through informative campaigns on behaviors, healthy practices, warning signs for the detection of COVID-19 [59,86], as well as encouraging vaccination and preventing misinformation about COVID-19 and its vaccine [85]. This information had to be updated and delivered from institutional sources, such as the MoH’s official website and social media (e.g., Facebook, Instagram) [71], as well as mass media (i.e., radio, tv, newspapers). Self-care and community care measures included the conduction of campaigns to promote a responsible self-medication for COVID-19 and other health conditions, as well as the prevention and reduction of stigma and discrimination against people with mental disorders [30,31,43,60,70]. To achieve these goals, policies stated that the participation of the community (i.e., leaders, volunteers, and community agents) should be included in the process of planning, organizing, monitoring, and evaluating mental health services [31,58].

To enhance mental health promotion activities, policies proposed working jointly and inter-institutionally, within institutions at the health system (e.g., health directorates, health centers, CMHCs) as well as those outside the health system (e.g., shelters, police and prosecutors, centers for young people and adolescents, and educational institutions) [31,43,46,56,58,87]. Finally, one policy proposed a series of research priorities to “prepare for the next pandemic”, including better communication in public health [88].

## Discussion

### Main findings

This study aimed to describe and analyze the mental health-related policies published by the Peruvian health authorities to address the population mental health needs and adequate health services during the COVID-19 pandemic. Policies and guidelines were retrieved from the two main health systems in the country: the MoH and EsSalud. One of the main documents published was the National Mental Health Plan [26], issued by the MoH, and two guidelines, one for the population and families affected by the COVID-19 pandemic, and the other directed to health workers. We found that these policies were used as a reference for institutions nationwide, including EsSalud and public hospitals, to design and implement their own mental health plans and programs.

Some policies were specifically designed to tend to the mental health of the Peruvian population, whereas others only had some contents related to mental health. As recommended by international guidelines [4–8], we identified guidelines directed towards mental health services, such as principles for the provision of mental health care, the strengthening of mental health services (e.g., allocation of budget, use of telemedicine), the detection of mental health problems, and the provision of specialized and non-specialized mental health treatments; as well as guidelines for the community outside the health system which could positively impact on their mental health (e.g., prevent misinformation, promotion of self-care, and delivery of PPEs for the population). Most policies, however, mainly focused on the detection and treatment of mental health issues instead of initiatives on promotion and prevention.

The analyzed policies were, in general, aligned to recommended guidelines worldwide [4–8,89,90].The most salient actions identified in these policies were the strengthening of their health systems by increasing the budget allocated for mental health, the expansion of telemedicine services, and the prioritization of “vulnerable populations”, such as health workers and institutionalized people, all of these relevant to address the challenges brought by the COVID-19 pandemic [91]. Moreover, we identified that the Peruvian response was consistent with international recommendations by adopting cross-cutting approaches such as human rights, gender, and interculturality. However, their adequacy was found to be partial, as the framework leaned heavily toward clinical detection and treatment, leaving a significant gap in community-based promotion and prevention strategies, which are considered central to the community mental health model [6,92].

In regards of the provision of mental health care, we identified specific contents that differed according to each population, for example, more complex interventions for people with mental disorders, such as psychotherapy and medications; in contrast to psychosocial support and non-pharmacological treatments for non-clinical populations, such as people with COVID-19 that experienced mental health issues or health workers. These specifications must have been useful for health workers when trying to decide what treatment should be provided.

Among good practices, we found the use of evidence to support the design of policies in EsSalud. This process enabled them to include specific contents and procedures on how to implement their recommendations, which might ease their use in health services’ routine practices [93]. This was a clear contrast to the policies from the MoH, which were broader and unspecific in their guidelines, and thereby, probably more difficult to implement in the quotidian routines of health facilities. Furthermore, in exploring the content of these policies, we identified a critical lack of specific budget allocation details within the individual guidelines. While the National Mental Health Plan mentions general funding, the sub-policies often lack the financial roadmap necessary for local execution. This observed incompleteness suggests that while the Peruvian response was evidence-aligned in principle, it remained structurally fragile in terms of guaranteed resources for front-line implementation.

Another difference between health systems was that the policies from EsSalud were mainly focused on attending the mental health of people with COVID-19 and their health workers; whereas the policies from the MoH focused on these populations and in the mental health impact of the pandemic in the general population, and to adequate the mental health services for people with mental disorders. Additionally, the reviewed policies included some approaches to support the provision of mental health services during the pandemic, which were aligned to international guidelines [4–8,89,90], such as human rights, gender, disability, and interculturality. Most policies also prioritized specific vulnerable populations, such as children, adolescents, women, older adults, LGTB community, and ethnic minorities, as recommended for policies on mental health [26].

Among policies’ weaknesses, we identified that several guidelines mentioned the transition from in-person to remote care [26,31], but no specific policy or guidance was found on how to adapt the current services to the use of technology, such as how to perform a virtual consultation or how to conduct long-distance home visits, which would be of used to ease the implementation of these policies [7,90]. Indeed, previous local studies showed that training and supervision of both health providers and patients were essential to effectively implement new technologies in public health systems, such as screening apps [94] and low-intensity digital treatments [95].

Likewise, the reviewed policies paid little attention to the monitoring and continuity of care for people with COVID-19 and mental health issues. Most policies focused on detecting and treating mental health issues, but not on conducting follow-ups or assuring their continuity of care, which has been recommended for long-term conditions, such as mental disorders [7,8]. Another weakness is that we only found some mentions to tackle abandonment and abuse of people with mental disorders but not specific actions on how to do it, despite being highly important in the human rights approach in which the mental health policies are based [5,8,26]. Finally, most policies lacked indicators to assess their implementation, which could be an important deficiency when evaluating the impact of their guidelines [96]. Such indicators might focus not only on coverage (i.e., how many people needing care received it) but also on the quality of care, including their effectiveness, safety and responsiveness [97].

### Comparison with other studies

Other countries, those from Europe [98] and the Asia-Pacific Region [99] conducted similar studies to analyze the mental health policies issued by their governments in face of the COVID-19 pandemic. In the Latin American region, countries such as Chile [9], Brazil [100] and Colombia [101] also prioritized the expansion of tele-mental health and the protection of front-line workers, aligned with WHO recommendations to maintain essential services [102]. However, like the Peruvian experience, these countries faced significant challenges regarding the integration of mental health into the general health response and the lack of robust monitoring frameworks.

Many of these countries also published general guidelines and plans to attend the mental health of their population, prioritizing specific groups, such as people with COVID-19 and their relatives -including those in home quarantine and hospitalized-, people with existing mental disorders, and health workers. A relevant absence on the Peruvian guidelines was the importance of involving people with mental disorders, which was considered a central component of policies in the European and Asian regions [98] and was a key recommendation by WHO to ensure person-centered care [103].

Unlike other low- and middle-income countries, such as Kenya [11] or countries in Sub-Saharan Africa [104], Peru had a prompt response to the COVID-19 pandemic in terms of attending the mental health of its population and preparing its mental health system.

We found that two months after the beginning of the pandemic, Peruvian policies proposed the preparation of the public health system infrastructure to contain its mental health impact, especially by moving from face-to-face care to remote care, as most countries in the Americas did [17]. Although Peru already had some policies related to the use of telemedicine, its use was limited, and the pandemic accelerated its utilization in mental health services [105,106] which is an important step towards innovations in public health services.

This prompt policy framework likely facilitated a significant quantitative expansion of services, for instance, mental health consultations in Peru increased from 400 000 to more than one million [107]. This trend is further supported by a time-series analysis conducted at 58 CMHCs, which demonstrated that despite an initial drop in unique users at the start of the outbreak, the system managed to recover its pre-pandemic care levels in approximately nine months. This recovery suggests a high level of institutional resilience, with the provision of health appointments and care activities continuing to grow despite the emergency [108].

However, this quantitative expansion and the resilience of the system must be balanced against the quality of the service experience from the users’ perspective. A qualitative study conducted in Peru [109] indicates that while patients and their families generally accepted tele-health as a necessary tool to maintain continuity of care, the transition imposed a new set of challenges that policies did not fully address. Beyond the technical difficulties, users reported that the effectiveness of remote consultations was often undermined by environmental and economic factors, such as the lack of privacy at home to discuss sensitive issues and the high cost of mobile data required for video calls [109].

Another qualitative study conducted with front-line health workers in Peru revealed a significant gap between these official regulations and their practical implementation [110]. While the policies mandated a swift transition to telemedicine, workers at CMHCs reported that the response was hindered by severe structural barriers, including a lack of institutional technological infrastructure and a digital literacy gap between providers and users. Furthermore, despite policies emphasizing community-based promotion, front-line reports indicated that these services were often suspended during the first wave to prioritize clinical emergencies, highlighting that mental health policies in emergency contexts must provide practical and flexible guidelines that account for the specific resource-limited characteristics of the local health system [110].

Furthermore, another local study showed that the perceived success of these interventions relied heavily on the emotional bond and the sense of ‘accompaniment’ provided by the staff, which many users of Peruvian CMHCs valued more than the modality itself [111]. Nevertheless, stakeholders noted that this continuity often depended on non-official platforms to reach patients, which reinforces the findings of our scoping review regarding the absence of standardized protocols for high-quality virtual care [111].

This evaluative analysis highlights a clear ‘policy-to-practice gap.’ The absence of the specific procedures and resources identified in our review explains why, in practice, the quantitative resilience of the Peruvian system was not necessarily supported by a complete policy design, but rather by the informal adaptations of its frontline staff.

### Policy considerations

This study integrates and summarizes the contents of different policies addressing the mental health of the Peruvian population. This will be useful for policymakers who plan future policies and identify the unattended topics that should be prioritized. These future policies, in contrast to the current ones, should include a set of indicators and monitoring procedures to assess their impact and make the necessary adjustments to assure their intended goals. It is well-known that data is crucial to understand the current context and changes and plan a corresponding service response [95]. Indeed, policies from high-income countries, such as Australia and New Zealand highlighted the importance of collecting data to monitor their policies, as Norway and Finland already do with their strong monitoring systems in place [95]. Specifically, monitoring should shift from solely tracking service volume, which showed resilience during the pandemic, to incorporating quality indicators such as user privacy, clinical outcomes, and the equitable reach of digital interventions.

Another relevant finding is the progress done in using telemedicine in public health systems to provide remote mental health care. It would be highly recommended to continue the use of technologies and reinforce the technological infrastructure of health centers nationwide. Other studies found that before the pandemic, health services in Peru used little technological interventions and main challenges were the lack of devices, poor internet connection, insufficient training for health workers, and patients difficulties to use technology [112]. To overcome these barriers, future policies should prioritize the institutionalization of technological resources, ensuring centers are equipped with official devices and stable connectivity, and provide practical, flexible operational guidelines that account for the socio-economic constraints of users, such as the high cost of mobile data or limited digital literacy. Implementing these practical recommendations will help transition from a reactive emergency response to a sustainable and inclusive hybrid care model [109–111].

### Strengths and limitations

This study integrates and summarizes the contents of different policies addressing the mental health of the Peruvian population issued by the two main health systems in Peru (the MoH and EsSalud) and published throughout the COVID-19 pandemic. This will be useful for policymakers who plan future policies to identify the unattended topics that should be prioritized. One limitation of this study is that all data was retrieved from non-academic platforms (e.g., www.gob.pe), which are government sources that lack the appropriate tools to perform a systematic search. This could limit its replicability by other authors and affect the accuracy of the search procedures (e.g., do not show a document that would meet inclusion criteria). To overcome this issue, we performed the search multiple times and by different people during the collection period trying to incorporate all eligible documents. Another limitation is that this study focused on the proposed policies and guidelines, but not on their actual implementation in real-life settings, which would require a different analysis. Nonetheless, the relevance of our study is that it systematizes the different topics covered by Peruvian policies, providing a general scope for other countries aiming to design similar policies.

Another limitation is that this study relied solely on policy documents from official sources, excluding unpublished or internal policy reports. This decision might have hindered relevant contents from policies that the Peruvian health authorities discussed but were not included in the final versions. Yet, as these contents did not reach the policy agenda, it was not deemed appropriate to include them in the analysis. Future research could include unpublished materials to have a broader comprehension of the policy process or involve relevant stakeholders for data validation and triangulation, as a previous study did by interviewing CMHC’s providers to collect their experiences applying these policies into their routine practices [110].

## Conclusions

During the COVID-19 pandemic, Peru designed several policies for the mental health of its population, including policies to be used within and outside the health system. These policies focused on the principles to consider in the design and implementation of mental health policies; the strengthening of the Peruvian healthcare system; the detection of mental disorders in prioritized populations; the provision of mental health care; and community-based strategies to attend to the mental health of the population. Peru had a rapid response to the pandemic, and this context accelerated the use of technology in its health services; however, some important weaknesses were found, such as the lack of specific procedures on how to implement the policies in real-life settings and the absence of indicators to assess their implementation, which could limit their effect.

For policymakers, these findings highlight that a successful crisis response requires moving beyond high-level mandates to providing specific, flexible operational guidelines that account for local resource limitations. Future regulations should prioritize the institutionalization of technological support and the establishment of robust monitoring systems based on quality indicators, such as user privacy and clinical outcomes, rather than just service volume. Reinforcing the current implementation of these policies requires a transition from emergency mandates to sustainable, well-monitored strategies. Indeed, it would be relevant for future studies to assess the role of other sectors during the COVID-19 pandemic, such as civil society organizations and private institutions.

The Peruvian experience provides valuable lessons for other low- and middle-income countries (LMICs) facing similar structural constraints. First, a rapid regulatory response must be accompanied by specific operational “roadmaps” to prevent a gap between national mandates and frontline capabilities. Second, the institutionalization of digital health requires a dual focus: providing infrastructure for providers while addressing the digital divide for users. Finally, LMICs should move toward person-centered policy designs that formally involve service users and civil society, ensuring that mental health systems are not only resilient in volume but also equitable and responsive to the population’s diverse needs during and after public health emergencies.

## Supporting information

S1 File PRISMA checklist. (PDF)

S2 File Search strategy. (DOCX)

S3 File Codebook. (DOCX)

S4 File Policies with contents related to improve the mental health of people during the COVID-19 outbreak. (DOCX)

S5 File Policies with contents related to the population’s general health with at least one mental health guideline. (DOCX)

S6 File Key definitions. (DOCX)
