# Supplementary material for: Scoping review of mental health-related policies issued in the context of the COVID-19 pandemic in Peru
Source: PLOS Ment Health. 2026 Apr 27;3(4):e0000459. doi: 10.1371/journal.pmen.0000459 (PMC13120698; doi:10.1371/journal.pmen.0000459)
Supplement: S1 File — (PDF) [file pmen.0000459.s001.pdf]

Table 1: PRISMA-ScR Checklist

Section	Item	PRISMA-ScR checklist item	Reported on page #
**Title**			
**Title**	1	Identify the report as a scoping review.	1
**Abstract**			
**Structured summary**	2	Provide a structured summary including, as applicable: background, objectives, eligibility criteria, sources of evidence, charting methods, results and conclusions that relate to the review question(s) and objective(s).	2-3
**Introduction**			
**Rationale**	3	Describe the rationale for the review in the context of what is already known. Explain why the review question(s)/objective(s) lend themselves to a scoping review approach.	4-5
**Objectives**	4	Provide an explicit statement of the question(s) and objective(s) being addressed with reference to their key elements (e.g., population or participants, concepts and context), or other relevant key elements used to conceptualize the review question(s) and/or objective(s)).	5
**Methods**			
**Protocol and registration**	5	Indicate if a review protocol exists, if and where it can be accessed (e.g., web address), and, if available, provide registration information including registration number.	6
**Eligibility criteria**	6	Specify the characteristics of the sources of evidence (e.g., years considered, language, publication status) used as criteria for eligibility, and provide a rationale.	7
**Information sources**	7	Describe all information sources (e.g., databases with dates of coverage, contact with authors to identify additional sources) in the search, as well as the date the most recent search was executed.	6
**Search**	8	Present the full electronic search strategy for at least one database, including any limits used, such that it could be repeated.	7 and 56 Supplementary Material 1
**Selection of sources of**	9	State the process for selecting sources of evidence (i.e., screening, eligibility) included	

Section	Item	PRISMA-ScR checklist item	Reported on page #
**evidence**		in the scoping review.	7-8
**Data charting process**	10	Describe the methods of charting data from the included sources of evidence (e.g., piloted forms; forms that have been tested by the team before their use, whether data charting was done independently, in duplicate) and any processes for obtaining and confirming data from investigators.	8
**Data items**	11	List and define all variables for which data were sought and any assumptions and simplifications made.	57 Supplementary Material 2
**Critical appraisal of individual sources of evidence**	12	***If done,*** provide a rationale for conducting a critical appraisal of included sources of evidence; describe the methods used and how this information was used in any data synthesis (if appropriate).	Not applicable
**Summary measures**	13	*Not applicable for scoping reviews.*	Not applicable
**Synthesis of results**	14	Describe the methods of handling and summarizing the data that were charted.	8
**Risk of bias across studies**	15	*Not applicable for scoping reviews.*	Not applicable
**Additional analyses**	16	*Not applicable for scoping reviews.*	Not applicable
**Results**			
**Selection of sources of evidence**	17	Give numbers of sources of evidence screened, assessed for eligibility, and included in the review, with reasons for exclusions at each stage, ideally using a flow diagram.	9
**Characteristics of sources of evidence**	18	For each source of evidence, present characteristics for which data were charted and provide the citations.	10 – 11
**Critical appraisal within sources of evidence**	19	***If done***, present data on critical appraisal of included sources of evidence (see item 12).	Not applicable
**Results of individual sources of evidence**	20	For each included source of evidence, present the relevant data that were charted that relate to the review question(s) and objective(s).	12 – 30
**Synthesis of**	21	Summarize and/or present the charting results as they relate to the review	31 – 39

Section	Item	PRISMA-ScR checklist item	Reported on page #
**results**		question(s) and objective(s).	
**Risk of bias across studies**	22	*Not applicable for scoping reviews.*	Not applicable
**Additional analyses**	23	*Not applicable for scoping reviews.*	Not applicable
**Discussion**			
**Summary of evidence**	24	Summarize the main results (including an overview of concepts, themes, and types of evidence available), explain how they relate to the review question(s) and objectives, and consider the relevance to key groups.	39 – 43
**Limitations**	25	Discuss the limitations of the scoping review process.	43
**Conclusions**	26	Provide a general interpretation of the results with respect to the review question(s) and objective(s), as well as potential implications and/or next steps.	44
**Funding**			
**Funding**	27	Describe sources of funding for the included sources of evidence, as well as sources of funding for the scoping review. Describe the role of the funders of the scoping review.	44

Mini-glossary of PRISMA-ScR terms

Charting – The process of data extraction in a scoping review is referred to as 'data charting', as per the Arksey and O'Malley (2005) and Levac et al. (2010) frameworks and the JBI guidance (2015, 2017).

Critical appraisal – Refers to the process of systematically examining research evidence to assess its validity, results and relevance before using it to inform a decision. This terminology is used for items 12 and 19, instead of 'risk of bias' (which is more applicable to systematic reviews of interventions) to be inclusive and acknowledge the various sources of evidence that may be included in a scoping review (e.g., quantitative and/or qualitative research, expert opinion, policy documents).

Information sources - This is where *sources of evidence* (see definition) are compiled from such as, bibliographic databases, social media platforms, websites, etc.

Sources of evidence – A more inclusive/ heterogeneous term is used to account for the fact that different types of evidence or data sources (e.g., quantitative and/or qualitative research, expert opinion, policy documents) may be eligible in a scoping review, as opposed to only studies. This is not to be confused with *information sources* (see definition).
