# Supplementary material for: Scoping review of mental health-related policies issued in the context of the COVID-19 pandemic in Peru
Source: PLOS Ment Health. 2026 Apr 27;3(4):e0000459. doi: 10.1371/journal.pmen.0000459 (PMC13120698; doi:10.1371/journal.pmen.0000459)
Supplement: S2 File — (DOCX) [file pmen.0000459.s002.docx]

**Supporting information 2. Search strategy**

| **Search strategy** | | **Results** |
| --- | --- | --- |
| LILACS | ("Mental Health" or "Mental Disorders" or "Mental problem*" or "Mental Disorder" or "Behavior Disorders" or Psych or "Mood disorder" or "Mood disorders" or "emotional problem" or distress or stress or schizop*) AND ("COVID 19" or "2019 novel coronavirus" or "coronavirus" or "coronavirus disease 2019" or "coronaviridae" or "2019-novel CoV" or "2019 ncov" or "ncov" or "novel cov" or "COVID 2019" or "COVID19" or "corona virus" or "nCoV-2019" or "nCoV2019" or "nCoV 2019" or "2019-ncov" or "2019ncov" or "COVID-19" or "Severe acute respiratory syndrome coronavirus 2" or "SARS-CoV-2" or "SARS2" or "SARS-CoV-19" or "sars cov2") AND peru* AND (year_cluster:[2020 TO 2023]) | 32 |
| Gray literature | | |
| Gobierno del Perú ([gob.pe/](https://www.gob.pe/)) | (“Mental health”, “COVID-19”) AND ( year_cluster [“Since 01-03-2020” AND “ Until 31-05-2023”]) AND (Content types_cluster [“Rules and legal documents”  AND “Reports and publications”]) | 8969 |
| The Social Security Health System (EsSalud) | In EsSalud, documents included were retrieved from the section on COVID-19 reports of its research institute: (<https://ietsi.essalud.gob.pe/reportes-covid-guias-practicas/>) | 42 |
