# Supplementary material for: Scoping review of mental health-related policies issued in the context of the COVID-19 pandemic in Peru
Source: PLOS Ment Health. 2026 Apr 27;3(4):e0000459. doi: 10.1371/journal.pmen.0000459 (PMC13120698; doi:10.1371/journal.pmen.0000459)
Supplement: S3 File — (DOCX) [file pmen.0000459.s003.docx]

**Supporting information 3. Codebook**

1. **General information**
   1. Document number
   2. Title
   3. Institution/Author
   4. # of Resolution (if applicable)
   5. Date published
   6. Type of document
   7. Link
2. **Policies for the General Population: Population/Universal initiatives**
   1. **Basic Services**
      1. Universal access to social interventions
      2. Access for people with mental disorders to social interventions
      3. Address social conditions that may increase COVID-19 risk
      4. Address barriers to care access
      5. Access to personal protective equipment
   2. **Security**
      1. Measures against human rights abuses
      2. Security for homeless/ness
      3. Actions to tackle abandonment/abuse
   3. **Strengthening community and family**
      1. Non-specialist psychosocial support to people undergoing overwhelming stress or trauma due to covid-19 restrictions or consequences
         1. Psychological First Aids
         2. Actions for grieving, bereavement
         3. Others
      2. Integrating mental health interventions into other sectors
      3. Communication and tackling fake news
         1. Disseminate up-to-date information about COVID-19
         2. Address misinformation, fake news and myths
   4. **Preparation and Coordination of the (Mental) Health System**
      1. Preparing infrastructure
         1. Physical infrastructure and materials of mental health services
         2. Digital infrastructure of mental health services: Transition from in-person to virtual and incorporating digital into in-person
         3. Measures to control COVID-19 in mental health services: Access to promotion and prevention, testing
      2. Training of providers
      3. Preparing the contents of mental health interventions
         1. What interventions are provided
         2. Contents adapted to mental health needs of specific populations (i.e. children, elderly)
3. **Policies for people with mental disorders**
   1. **Detection**
      1. How: Screening tools, who screens, modality, etc.
      2. For whom: Identification and monitoring for at risk population
         1. For health care workers
         2. For children, adolescents, antenatal and postnatal periods
         3. For older adults, including dementia, etc.
         4. For people with disabilities and preexisting mental disorders
         5. For people infected, in treatment or survivors of COVID-19
         6. For people who lost a beloved one
         7. For quarantined or isolated population
   2. **Care**
      1. Help-seeking
      2. Improving access and coverage to psychosocial interventions
         1. Remote
         2. In-person
   3. **Integrating mental health services into universal health coverage**
   4. **Eliminating coercion in mental health care**
      1. Human rights-based interventions
      2. Changes in law against dignity/ Human Rights
      3. Mis/use of mental health laws
   5. **For people with common mental disorders (i.e. anxiety, mild depression)**
      1. Transition to online delivery of psychoeducation
      2. Psychotherapy
      3. Psychopharmacology
   6. **For people with more severe, concurrent, or complex presentations of mental disorders**
      1. Reinforced essential in-person services
      2. Trained providers to minimize exposure to COVID-19
      3. No discontinuation of treatments
      4. For those in care homes
   7. **For all people with MD**
      1. Enhanced attention to physical health
      2. Protocols to deal with acute patients
      3. Monitoring people on long-term psychotropic medications
   8. **Sustainability of mental health care services**
      1. Funding
      2. Existing policies for access to mental health services
      3. Availability of psychotropic medication
      4. Policies to address redeployment of specialists
      5. Coordination and collaboration within the healthcare system
4. **Policies for people with COVID-19**
   1. **Training of MH providers in COVID-19 prevention and control**
   2. **COVID-19 Health services for people with MD**
      1. Detection of MH problems
      2. Assure acces with no discrimination
      3. Availability of hospital transfers from MH services
      4. Assure access to palliative/end of life care for people with MD
   3. **COVID 19-specific mental health services**
      1. Mild cases: Remote monitoring while being at home
      2. Moderate/Severe cases: Psychosocial support while hospitalized; family support
      3. Death: Grieving with loss
      4. Sequels/Long-term effects and/or Long COVID-19
   4. **Community interventions (no health system)**
5. **Policies for people delivering essential services**
   1. **For workers in general**
      1. Widely available psychosocial support
   2. **For heatlh workers**
      1. Psychosocial support
      2. Assessment
         1. Of mental health status
         2. Of drivers of mental ill-health
         3. Of consequences
      3. Specialist care
         1. To address exposure to trauma, overwhelming stressors
         2. For underlying mental or substance use conditions
      4. Protection of health workers (Including personal protective equipment)
6. **Summary**
7. **Additional information**
