# Supplementary material for: Scoping review of mental health-related policies issued in the context of the COVID-19 pandemic in Peru
Source: PLOS Ment Health. 2026 Apr 27;3(4):e0000459. doi: 10.1371/journal.pmen.0000459 (PMC13120698; doi:10.1371/journal.pmen.0000459)
Supplement: S4 File — (DOCX) [file pmen.0000459.s004.docx]

**Supporting information 4. Policies with contents related to improve the mental health of people during the COVID-19 outbreak.**

| **N** | **Title** | **Institution/ Author** | **Date** | **Content related to mental health** | **Scope** | **Link** |
| --- | --- | --- | --- | --- | --- | --- |
| **1** | **Recomendaciones clínicas para la prevención y manejo de problemas de salud mental en población en aislamiento por COVID 19** | EsSalud/ IETSI | 4/1/2020 | Recommendations for people in isolation, based on international and MoH guidelines | All health centers of EsSalud | <http://www.essalud.gob.pe/ietsi/pdfs/guias/reportes/Reporte_COVID_Nro_8.pdf> |
| **2** | **Recomendaciones clínicas para la prevención y manejo de problemas de salud mental en personal de salud en el marco de pandemia por COVID 19** | EsSalud/ IETSI | 4/1/2020 | Recommendations for the prevention and management of mental health problems in health personnel in the context of the pandemic | All health centers of EsSalud | <http://www.essalud.gob.pe/ietsi/pdfs/guias/reportes/Reporte_COVID_Nro_9.pdf> |
| **3** | **Guía técnica para el cuidado de la salud mental de la población afectada, familias y comunidad, en el contexto del COVID-19** | MoH/ Directorate of Mental Health | 4/1/2020 | Procedures for the care and self-care of mental health for general population and vulnerable groups | Nacional scope | <https://cdn.www.gob.pe/uploads/document/file/1226901/500120200811-2899800-17t0718.pdf?v=1597184393> |
| **4** | **Libro: Guía técnica para el cuidado de la salud mental del personal de la salud en el contexto del COVID-19** | MoH/ Directorate of Mental Health | 5/1/2020 | Guidelines for the care of the mental health of health workers | Nacional scope | <https://cdn.www.gob.pe/uploads/document/file/1226898/500020200811-2899800-prix6f.pdf> |
| **5** | **Guía técnica para el cuidado de la salud mental de la población afectada, familias y comunidad, en el contexto del COVID-19: Especificaciones para la atención de la salud mental de mujeres víctimas de la violencia de género** | MoH/ Directorate of Mental Health | 5/1/2020 | Guidelines for mental health care for women victims of gender-based violence. | Nacional scope | <https://cdn.www.gob.pe/uploads/document/file/1226896/500220200811-2899800-lu3dk6.pdf> |
| **6** | **Plan de implementación y supervisión de cuidado y autocuidado de salud mental del personal de salud del INEN en el contexto COVID-19** | National Institute of Neoplastic Diseases (NIND) | 5/4/2020 | Approval of the "Plan for Implementation and Supervision of Mental Health Care and Self-Care of Health Personnel of the National Institute of Neoplastic Diseases in the Covid-19 Context" | NIND | <https://portal.inen.sld.pe/wp-content/uploads/2020/05/RJ-145-2020.pdf> |
| **7** | **Directiva Sanitaria que establece disposiciones para brindar información y acompañamiento psicosocial a pacientes hospitalizados con infección por COVID-19 y sus familiares** | MoH/ General Directorate of Strategic Interventions in Public Health | 5/22/2020 | Guidelines for psychosocial support for people hospitalized for COVID-19 | All health centers with inpatient Health Service Providers | <https://cdn.www.gob.pe/uploads/document/file/729932/RM_312-2020-MINSA.PDF> |
| **8** | **Plan de salud mental en el contexto de COVID-19** | MoH/ Directorate of Mental Health | 6/6/2020 | Plan to promote the well-being of the population of Peru through mental health care in the context of COVID-19 | Nacional scope | <https://cdn.www.gob.pe/uploads/document/file/811138/Plan_de_Salud_mental_.PDF?v=1591547874> |
| **9** | **Aprobación del cuadro para asignación de personal provisional del Instituto Nacional de Salud Mental** | MoH/ National Institute of Mental Health | 8/4/2020 | Approval of budgets for the assignment of temporary staff of the National Institute of Mental Health (NIMH) "Honorio Delgado - Hideyo Noguchi" | NIMH “Honorio Delgado - Hideyo Noguchi” | <https://cdn.www.gob.pe/uploads/document/file/1221865/RESOLUCIO%CC%81N_SECRETARIAL_N_155-2020-MINSA.PDF> |
| **10** | **Conformación del Equipo de Salud Mental para el acompañamiento psicosocial a pacientes hospitalizados por COVID-19 y sus familiares del Hospital Nacional Hipólito Unanue** | Hipólito Unanue National Hospital (HUNH) | 9/9/2020 | Approve the formation of the Mental Health Team for the Psychosocial accompaniment of patients hospitalized by COVID-19 and their relatives at the HUNH. | Patients hospitalized by COVID-19 and their families at the HUNH. | <https://cdn.www.gob.pe/uploads/document/file/1299949/RD-189-09-2020.pdf> |
| **11** | **Guía técnica para el cuidado de la salud mental del personal de salud en el contexto COVID-19 del Instituto Nacional Materno Perinatal** | National Maternal Perinatal Institute | 9/23/2020 | Recommendations for the prevention and management of mental health problems in health personnel in the context of the pandemic | All staff of National Maternal Perinatal Institute | <https://cdn.www.gob.pe/uploads/document/file/1353639/RD%20N%C2%B0%20173-DG-INMP/MINSA.pdff> |
| **12** | **Plan de implementación y supervisión de cuidado y autocuidado de la salud mental de los pacientes oncológicos con COVID-19 y sus familiares en el Instituto Nacional de Enfermedades Neoplásicas** | National Institute of Neoplastic Diseases (NIND) | 10/6/2020 | Approval of the "Plan for Implementation and Supervision of Mental Health Care and Self-Care of oncology patients with Covid-19 and their families of the NIND in the Covid-19 context" | National Institute of Neoplastic Diseases (NIND) | <https://cdn.www.gob.pe/uploads/document/file/1497565/RJ%20307-2020-J-INEN.pdf.pdf> |
| **13** | **Aprobar el plan de prevención , atención y recuperación en salud mental a población general y en situación de riesgos frente a la pandemia y la segunda ola pandémica del covid-19 de la red de salud N° 04 Aguaytia - San Alejandro** | Regional Directorate of Health of Ucayali | 11/12/2020 | Approval of the mental health prevention, care and recovery plan for the general population at risk during the pandemic and the second wave of COVID-19, of the Health Network 04 Aguaytia - San Alejandro | All the staff of the Health Network Nº4 Aguaytya - San Alejandro | <https://www.gob.pe/institucion/regionucayali-risaguaytia/normas-legales/1466449-0299-2020-gru-diresa-drsasa-de> |
| **14** | **Resolución Directoral N° 1490-2020-GRH/DIRESA Huánuco** | Regional Directorate of Health of Huánuco | 12/3/2020 | Approve the local intersectoral committee between women's emergency centers, first-level health facilities, and CMHC. | All Women's Emergency Center (WEC) and CMHC | <http://www.diresahuanuco.gob.pe/portal/publicaciones/resoluciones/RD2020/SALUD%20MENTAL/R.D.%20ACTUACI%C3%93N%20CONJUNTA%20ENTRE%20CEM%20Y%20EESS%20-%20HU%C3%81NUCO.pdf> |
| **15** | **Resolución Directoral N° 179-2021-GRH-DIRESA Huánuco** | Regional Directorate of Health of Huánuco | 3/4/2021 | Approval of the Clinical Psychosocial Support Plan for 2021 of "Pakkarin" CMHC | The CMHC Pakkarin of Huánuco | <http://www.diresahuanuco.gob.pe/portal/publicaciones/resoluciones/RD2021/R.D_Nro_179-2021-GRH-DIRESA.pdf> |
| **16** | **Resolución Directoral N° 178-2021-GRH-DIRESA Huánuco** | Regional Directorate of Health of Huánuco | 3/4/2021 | Approval of the Clinical Psychosocial Support Plan for 2021 of "Esperanza" CMHC | The CMHC Esperanza of Huánuco | <http://www.diresahuanuco.gob.pe/portal/publicaciones/resoluciones/RD2021/R.D_Nro_178-2021-GRH-DIRESA.pdf> |
| **17** | **Resolución Directoral N° 177-2021-GRH-DIRESA Huánuco** | Regional Directorate of Health of Huánuco | 3/4/2021 | Approval of the Clinical Psychosocial Support Plan for 2021 of "Grover Mori Romero" CMHC | The CMHC Grover Mori Romero of Huánuco | <http://www.diresahuanuco.gob.pe/portal/publicaciones/resoluciones/RD2021/R.D_Nro_177-2021-GRH-DIRESA.pdf> |
| **18** | **Resolución Directoral N° 176-2021-GRH-DIRESA Huánuco** | Regional Directorate of Health of Huánuco | 3/4/2021 | Approval of the Clinical Psychosocial Support Plan for 2021 of "Bella Durmiente" CMHC | The CMHC Bella Durmiente of Huánuco | <http://www.diresahuanuco.gob.pe/portal/publicaciones/resoluciones/RD2021/R.D_Nro_176-2021-GRH-DIRESA.pdf> |
| **19** | **Resolución Directoral N° 175-2021-GRH-DIRESA Huánuco** | Regional Directorate of Health of Huánuco | 3/4/2021 | Approval of the Clinical Psychosocial Support Plan for 2021 of "Kushisha Panatahua" CMHC | The CMHC Kushisha Patanahua of Huánuco | <http://www.diresahuanuco.gob.pe/portal/publicaciones/resoluciones/RD2021/R.D_Nro_175-2021-GRH-DIRESA.pdf> |
| **20** | **Resolución Directoral N° 174-2021-GRH-DIRESA Huánuco** | Regional Directorate of Health of Huánuco | 3/4/2021 | Approval of the Clinical Psychosocial Support Plan for 2021 of "Chinchaysuyo" CMHC | The CMHC Chinchaysuyo of Huánuco | <http://www.diresahuanuco.gob.pe/portal/publicaciones/resoluciones/RD2021/R.D_Nro_174-2021-GRH-DIRESA.pdf> |
| **21** | **Protocolo para la vacunación a personas con trastorno mental grave y trastornos del neurodesarrollo** | MoH/ General Directorate of Strategic Interventions in Public Health | 5/20/2021 | Guidelines for vaccination of people with severe mental disorders and neurodevelopmental disorders, who were considered a vulnerable population. | Nacional scope | <https://cdn.www.gob.pe/uploads/document/file/1903754/Protocolo.pdf?v=1621561275> |
| **22** | **Documento técnico: Orientaciones técnica para el cuidado integral de la salud mental de la población adolescente** | MoH/ Directorate of Mental Health | 6/13/2021 | Establish guidelines for health personnel who provide comprehensive mental health care to the adolescent population that is cared for in establishments nationwide | Nacional scope | [https://cdn.www.gob.pe/uploads/document/file/1944836/Documento%20Técnico%20-Orientaciones%20Técnicas%20para%20el%20Cuidado%20integral%20de%20la%20Salud%20Mental%20de%20la%20Población%20Adolescente.pdf](https://cdn.www.gob.pe/uploads/document/file/1944836/Documento%20T%C3%A9cnico%20-Orientaciones%20T%C3%A9cnicas%20para%20el%20Cuidado%20integral%20de%20la%20Salud%20Mental%20de%20la%20Poblaci%C3%B3n%20Adolescente.pdf) |
| **23** | **Teleconferencia: Programación de productos farmacéuticos y dispositivos médicos estratégicos en salud mental para el año 2021** | MoH/ General Directorate of Strategic Interventions in Public Health |  | Criteria for programming mental health medications and those responsible for supply | National scope | <https://cdn.www.gob.pe/uploads/document/file/1033706/2021_-_09_CRITERIOS_DE_PROGRAMACION_DE_SALUD_MENTAL.pdf> |
| **24** | **Guía técnica para el cuidado de la salud mental de mujeres en situación de violencia ocasionada por la pareja o expareja** | MoH/ Directorate of Mental Health | 6/22/2022 | Guidelines for mental health care for women victims of gender-based violence. | National scope | <https://www.gob.pe/institucion/minsa/informes-publicaciones/3149441-guia-tecnica-para-el-cuidado-de-la-salud-mental-de-mujeres-en-situacion-de-violencia-ocasionada-por-la-pareja-o-expareja> |
| **25** | **Pautas para la prevención del suicidio en mi comunidad** | MoH/ Directorate of Mental Health | 8/15/2022 | Guidelines for the prevention of suicide in community settings | National scope | [https://cdn.www.gob.pe/uploads/document/file/3501367/Pautas%20para%20la%20prevención%20del%20suicido%20en%20mi%20comunidad.pdf?v=1660580499](https://cdn.www.gob.pe/uploads/document/file/3501367/Pautas%20para%20la%20prevenci%C3%B3n%20del%20suicido%20en%20mi%20comunidad.pdf?v=1660580499) |

Note: CMHC = Community Mental Health Centers; MoH = Ministry of Health; WEC = Women's Emergency Center; Guidelines = Detailed document that specifies the contents and methods to implement certain intervention; Plan = Broader management tool that stablishes goals and strategies to achieve certain aim.
