# Supplementary material for: Scoping review of mental health-related policies issued in the context of the COVID-19 pandemic in Peru
Source: PLOS Ment Health. 2026 Apr 27;3(4):e0000459. doi: 10.1371/journal.pmen.0000459 (PMC13120698; doi:10.1371/journal.pmen.0000459)
Supplement: S5 File — (DOCX) [file pmen.0000459.s005.docx]

**Supporting information 5. Policies with contents related to the population’s general health with at least one mental health guideline.**

| **N** | **Title** | **Institution/ Author** | **Date** | **Content related to mental health** | **Field of application** | **Link** |
| --- | --- | --- | --- | --- | --- | --- |
| 1 | **Recomendaciones para el aislamiento domiciliario en contexto de pandemia por COVID 19** | EsSalud/ IETSI | 3/1/2020 | Recommendations to establish routines and maintain contact with close people during home isolation | All health centers of EsSalud | <http://www.essalud.gob.pe/ietsi/pdfs/guias/reportes/Reporte_COVID_Nro_2.pdf> |
| 2 | **Recomendaciones clínicas para el manejo del parto en el contexto de pandemia de COVID 19** | EsSalud/ IETSI | 4/1/2020 | Recommendations for the management of labor in pregnant women with suspected or confirmation of COVID-19 infection | All health centers of EsSalud | <http://www.essalud.gob.pe/ietsi/pdfs/guias/reportes/Reporte_COVID_Nro_11.pdf> |
| 3 | **Lineamientos que refuerzan el cuidado integral de salud en el primer nivel de atención en el contexto de la pandemia COVID-19** | MoH/ General Directorate of Strategic Interventions in Public Health | 4/8/2020 | Guidelines for health care at the primary level including mental health | National scope | <https://cdn.www.gob.pe/uploads/document/file/581620/RM_182-2020-MINSA_Y_ANEXOS.PDF?v=1586384623> |
| 4 | **Directiva Sanitaria para la prevención y atención de la gestante y el recién nacido con riesgo o infección por COVID-2019** | MoH/ Directorate of Sexual and Reproductive Health | 4/30/2020 | Guidelines for mental and psychosocial health care for mothers and relatives with COVID-19 | National scope | <https://cdn.www.gob.pe/uploads/document/file/671164/RM_245-2020-MINSA.PDF> |
| 5 | **Lineamientos para el fortalecimiento de acciones en establecimiento de salud, redes de salud** | MoH/ General Directorate of Disaster Risk Management and National Defense in Health | 5/6/2020 | Guidelines for the development of health interventions to monitor and care for COVID-19 patients and their families or contacts | National scope | <https://cdn.www.gob.pe/uploads/document/file/686702/R.M._N__255-2020-MINSA.PDF> |
| 6 | **Lineamientos para la vigilancia de la salud de los trabajadores con riesgo de exposición a COVID-19** | MoH/ General Directorate of Strategic Interventions in Public Health | 5/8/2020 | Request to include mental health measures within the worker's health surveillance plan in the context of COVID-19 | National scope | <https://cdn.www.gob.pe/uploads/document/file/695454/resolucion-ministerial-n-265-2020-minsa.pdf?v=1588995651> |
| 7 | **Resolución Ministerial N° 263-2020-MoH** | MoH/ General Directorate of Strategic Interventions in Public Health | 5/8/2020 | Guidelines for the development of processes, records and access to information to guarantee the follow-up and care of patients with COVID-19 | Nacional scope | <https://cdn.www.gob.pe/uploads/document/file/693958/RM_263-2020-MINSA.PDF?v=1588944924> |
| 8 | **Norma técnica para la adecuación del primer nivel de atención en salud durante la epidemia por COVID-19** | MoH/ General Directorate of Strategic Interventions in Public Health | 5/21/2020 | Guidelines for the adaptation and implementation of the articulated and integrated organization in health center services | National scope | <https://cdn.www.gob.pe/uploads/document/file/733623/NTS_160-2020-MINSA_Adecuacion_SS_PNAS.pdf?v=1590469529> |
| 9 | **Plan de intervención para Comunidades Indígenas y Centros Poblados Rurales de la Amazonía frente a la emergencia del COVID-19** | MoH/ Directorate of Indigenous and Native Peoples | 5/21/2020 | Guidelines to contribute to the reduction and containment of the health, the social, and economic impact of the COVID-19 pandemic on indigenous communities and rural areas in the Amazon. | All public health centers which attend indigenous communities and rural population centers in the Peruvian Amazon. | <https://cdn.www.gob.pe/uploads/document/file/733621/RM_308-2020-MINSA.PDF?v=1590469150> |
| 10 | **Documento Técnico: Lineamientos para el fortalecimiento de acciones de respuesta en establecimientos de salud, redes de salud y oferta móvil frente al COVID-19 (en fase de transmisión comunitaria)** | MoH/ General Directorate of Disaster Risk Management and National Defense in Health | 5/22/2020 | Guidelines for strengthening response actions in health establishments, health networks, and mobile offers against COVID-19 | National scope | <https://cdn.www.gob.pe/uploads/document/file/729861/RM_309-2020-MINSA.PDF> |
| 11 | **Lineamientos para el retorno progresivo a las actividades laborales en el contexto de pandemia por COVID 19** | EsSalud/ IETSI | 6/1/2020 | General guidelines of preventive measures and practices for the prevention of contagion of COVID - 19 in work centers | All workplaces | <http://www.essalud.gob.pe/ietsi/pdfs/guias/reportes/Reporte_COVID_Nro_21.pdf> |
| 12 | **Directiva para la reincorporación progresiva de la actividad física y de recreación en espacios públicos** | MoH/ General Directorate of Strategic Interventions in Public Health | 6/2/2020 | Recommendations to strengthen the mental and physical health of the population through the practice of physical and recreational activity, maintaining measures to prevent community transmission of COVID-19 | Nacional scope | <https://cdn.www.gob.pe/uploads/document/file/770868/RM_350.pdf?v=1591304016> |
| 13 | **Consideraciones éticas para la toma de decisiones en los servicios de salud durante la pandemia Covid-19** | MoH/ Working Group on Bioethical Aspects | 6/24/2020 | Guidelines and ethical considerations for the care of COVID-19 patients and their families | Nacional scope | <https://cdn.www.gob.pe/uploads/document/file/871496/consideraciones-eticas-para-la-toma-de-decisiones-en-los-servicios-de-salud-durante-la-pandemia-covid-19-v2.pdf?v=1676878128> |
| 14 | **Recomendaciones clínicas para el manejo de pacientes geriátricos en casas de reposo durante la pandemia por COVID 19** | EsSalud/ IETSI | 7/1/2020 | Clinical recommendations for the prevention, diagnosis, and management of geriatric patients. | All nursing homes, residences or shelters that house older adults | <http://www.essalud.gob.pe/ietsi/pdfs/guias/reportes/Reporte_COVID_Nro_22.pdf> |
| 15 | **Directiva para la entrega de información al paciente hospitalizado con infección por COVID-19 y a sus familiares en el Hospital de Emergencia Ate Vitarte** | General Directorate of the Ate Vitarte Emergency Hospital | 7/17/2020 | Provisions, procedures, and communication channels to provide information and psychosocial support to hospitalized patients with COVID-19 infection and their authorized family members. | All staff of the Emergency Hospital Ate Vitarte | <https://cdn.www.gob.pe/uploads/document/file/1201017/RD_051.pdf> |
| 16 | **Acuerdo Regional N°000131-CR.LAMB/CR** | Lambayeque Government | 7/26/2020 | Request for improvements in the communication protocol with the relatives of COVID-19 patients in the health services in the Lambayeque Region | Regional Government of Health, the Regional Operations Command COVID-19 and the Essalud Hospitals of Lambayeque | <https://www.gob.pe/institucion/regionlambayeque/normas-legales/1139006-000131-gr-lamb-cr> |
| 17 | **Plan Operativo Institucional (POI) Multianual 2021 – 2023** | MoH/ National Center for Strategic Planning | 7/31/2020 | Approve the Multiannual Institutional Operational Plan 2021 - 2023 and the budget allocation for the MoH (including mental health) | Nacional scope | <https://cdn.www.gob.pe/uploads/document/file/1204064/plan-operativo-institucional-poi-multianual-2021-2023-del-ministerio-de-salud.pdf?v=1596590979> |
| 18 | **Recomendaciones clínicas para cuidados paliativos en pacientes con COVID 19 hospitalizados en áreas no UCI** | EsSalud/ IETSI | 8/1/2020 | Medidas para cuidados paliativos y acompañamiento a familiares. Además, pautas de autocuidado para trabajadores de salud | All hospitals which manage COVID-19 patients in non UCI areas | <http://www.essalud.gob.pe/ietsi/pdfs/guias/reportes/Reporte_COVID_Nro_23.pdf> |
| 19 | **Documento Técnico: Medidas para el cuidado y prevención frente a la COVID-19 en centros de atención residencial de personas adultas mayores** | MoH/ General Directorate of Strategic Interventions in Public Health | 8/28/2020 | Approve the Technical Document: Measures for care and prevention against COVID-19 in residential care centers for the elderly | All public and private residential care facilities of elderly people | <https://cdn.www.gob.pe/uploads/document/file/1273730/DOC.%20TECNICO.pdf> |
| 20 | **Resolución Directoral N° 689-2020-GRA/GG-GRDS-DIRESA-DA** | Central Office of the Regional Department of Health of Ayacucho | 8/31/2020 | Provides guidelines on biosafety measures in health center services | Central Office of the Regional Department of Health of Ayacucho | <https://www.saludayacucho.gob.pe/diresadocs_/contenido_/Documentos/Documentos_gestion/Plan%20vigilancia%20y%20control%20de%20COVID19.pdf> |
| 21 | **Resolución Ministerial N° 678-2020-MoH** | MoH/ General Office of Planning, Budget, and Modernization | 9/1/2020 | Approval of the list of Goods and Services in the Plan of Action-Surveillance, Containment, and Care of Cases of the new COVID-19 in Peru (Includes mental health), and assignation presupuestal for mental health care. | National Scope | [https://cdn.www.gob.pe/uploads/document/file/1276311/Resolución%20Ministerial%20N°%20678-2020-MINSA.PDF?v=1598977632](https://cdn.www.gob.pe/uploads/document/file/1276311/Resoluci%C3%B3n%20Ministerial%20N%C2%B0%20678-2020-MINSA.PDF?v=1598977632) |
| 22 | **Modificatoria del Plan de Estrategia Publicitaria 2020** | MoH/ Executive Directorate of Health Promotion | 9/10/2020 | Approve the modification of the 2020 Advertising Strategy Plan of the HoH, incorporating the Campaigns "Let's not lower our guard", "Eat healthily, live healthily" and "Healthy coexistence" | Nationally via mass and alternative communication media | [https://cdn.www.gob.pe/uploads/document/file/1298510/Resolución%20Ministerial%20N°%20716-2020-MINSA.PDF](https://cdn.www.gob.pe/uploads/document/file/1298510/Resoluci%C3%B3n%20Ministerial%20N%C2%B0%20716-2020-MINSA.PDF) |
| 23 | **Plan de respuesta frente al coronavirus covid19 por alerta roja** | Teaching Hospital (HD) “Mother Child San Bartolomé | 10/13/2020 | Guidelines to deal with the health emergency through the Front Plan Response to COVID-19 by Red Alert | HD "Madre Niño San Bartolomé". | <https://cdn.www.gob.pe/uploads/document/file/1410626/RD%20144%20SB%202020.pdf.pdf?v=1604015997> |
| 24 | **Documento Técnico: Plan de preparación y respuesta ante posible segunda ola pandémica por COVID-19 en el Perú** | MoH/ General Directorate of Disaster Risk Management and National Defense in Health | 11/9/2020 | Guidelines to improve the preparedness and response capacity of the Health Sector to reduce the impact of morbidity and mortality from COVID-19 in the Peruvian population | All the organic units of MoH; and referential for public, private or mixed health institutions. | <https://cdn.www.gob.pe/uploads/document/file/1437467/RM%20N%C2%B0928-2020-MINSA.pdf.pdf> |
| 25 | **Plan de Preparación y Respuesta ante la Posible Segunda Ola Pandémica por COVID-19 en la Región Cusco 2020-2021** | Regional Directorate of Health of Cuzco | 11/2020 | Guidelines to improve the preparedness and response capacity of the Health Sector to reduce the impact of morbidity and mortality from COVID-19 in the Cusco Region | All health centers of Cusco | <http://www.diresacusco.gob.pe/comunica_covid19/plan2daolacovid.pdf> |
| 26 | **Directiva sanitaria para garantizar la salud de las gestantes y la continuidad de la atención en planificación familiar ante la infección por COVID-19** | MoH/ Directorate of Sexual and Reproductive Health | 12/2/2020 | Provides mental health care within the guidelines for the intervention of pregnant women, in family planning and victims of physical and sexual abuse | National Scope | <https://cdn.www.gob.pe/uploads/document/file/1865053/5212.pdf> |
| 27 | **Guía de Práctica Clínica sobre intervenciones de mantenimiento de Salud para Adultos Mayores en el Primer Nivel de Atención** | EsSalud/ IETSI | 12/1/2020 | Recommendations for the care of the elderly in the first level of attention | All health services available at the first level care of Essalud. | <http://www.essalud.gob.pe/ietsi/pdfs/tecnologias_sanitarias/GPC_Geriatria_Version_Corta.pdf> |
| 28 | **Resolución Directoral N° 311-2020-HNHU-DG** | Hipólito Unanue National Hospital (HUNH) | 12/14/2020 | Approval of a care plan for COVID and non-COVID services | HUNH | <https://cdn.www.gob.pe/uploads/document/file/1561170/RD-311-12-2020.pdf.pdf> |
| 29 | **Directiva Sanitaria que establece el paquete básico para el cuidado integral de la salud de adolescentes en el contexto del COVID-19** | MoH/ General Directorate of Strategic Interventions in Public Health | 12/18/2020 | Provides screening guidelines for mental disorders, mental health consulting services, educational sessions for families on mental health | Nacional scope | <https://cdn.www.gob.pe/uploads/document/file/1487125/Resoluci%C3%B3n%20Ministerial%20N%C2%B01046-2020-MINSA..PDF> |
| 30 | **Resolución Directoral N° 352-2020-HNHU-DG** | Hipólito Unanue National Hospital (HUNH) | 12/30/2020 | Approval of the psychosocial and communication support plan for patients hospitalized for COVID-19 | HUNH | <https://cdn.www.gob.pe/uploads/document/file/1548193/R.D.N%C2%B0352-2020-HNHU-DG%20PLAN%20DE%20ACOMPA%C3%91AMIENTO%20PSICOSOCIAL.pdf.pdf> |
| 31 | **Norma Técnica de Salud para la Adecuación de los Servicios de Salud del Primer Nivel de Atención de Salud frente a la pandemia por COVID-19 en el Perú** | MoH/ General Directorate of Health Insurance and Health Exchange | 1/7/2021 | Guidelines for the promotion of self-care and mental health care in health interventions, and promotion of the participation of social organizations to strengthen health interventions | All health centers of first level | [https://cdn.www.gob.pe/uploads/document/file/1535219/Norma%20Técnica%20de%20Salud%20N°171-MINSA-2021-DGAIN.pdf](https://cdn.www.gob.pe/uploads/document/file/1535219/Norma%20T%C3%A9cnica%20de%20Salud%20N%C2%B0171-MINSA-2021-DGAIN.pdf) |
| 32 | **Convenio 096-2020-MoH** | MoH/ General Directorate of Health Personnel | 1/13/2021 | Agreement between the MoH and the Universidad Peruana Antenor Orrego for university health students and professionals to support clinical follow-up and health promotion activities during the pandemic | Directions of the Integrated Health Networks (DIHN) in regions with high prevalence of the COVID-19 pandemic | <https://cdn.www.gob.pe/uploads/document/file/1542642/Convenio%20N%C2%B0096-2020-MINSA.PDF> |
| 33 | **Convenio 001-2021-MoH** | MoH/ Directorate of Mental Health | 1/14/2021 | Agreement to promote mental health in vulnerable populations of the Peruvian Amazon | All CMHC and mental health services of hospitals | <https://cdn.www.gob.pe/uploads/document/file/1545937/CONVENIO%20N%C2%B0%20001-2021-MINSA.pdf> |
| 34 | **Resolución Ministerial N° 102-2021-MoH. Campaña de salud mental: “Convivencia saludable”** | MoH/ General Office of Human Resources Management | 1/27/2021 | Contracting of media for the dissemination of the mental health campaign "healthy coexistence" | Nationally via mass and alternative communication media | <https://cdn.www.gob.pe/uploads/document/file/1594750/Resoluci%C3%B3n%20Ministerial%20%20N%C2%B0%20102-2021-MINSA.pdf> |
| 35 | **Norma Técnica de Salud para la atención de salud ambulatoria, quirúrgica electiva, en hospitalización y servicios médicos de apoyo, frente a la pandemia por COVID-19 en el Perú** | MoH/ General Directorate of Health Insurance and Health Exchange | 1/29/2021 | Provides guidelines on biosafety measures in health center services | Nacional scope | <https://cdn.www.gob.pe/uploads/document/file/1616908/Resoluci%C3%B3n%20Ministerial%20N%C2%B0107-2021-MINSA.PDF> |
| 36 | **Directiva Administrativa que establece lineamientos para la aplicación de lo dispuesto en el artículo 4 del Decreto de Urgencia Nº012-2021** | MoH/ General Office of Human Resources Management | 2/16/2021 | Establishes the guidelines for making budgetary modifications, including the budget for the Mental Health Control and Prevention Program. | All implementing units of the Health Sector | <https://cdn.www.gob.pe/uploads/document/file/1677804/Directiva%20Administrativa%20N%C2%BA305-2021-MINSA-OGPPM.pdf> |
| 37 | **Derecho de los adolescentes al paquete básico de atención integral de salud en el contexto de la COVID-19. Rotafolio dirigido al personal de salud que atiende a la población adolescente** | MoH/ Directorate of Life and Integral Care | 3/1/2021 | Provides the content that will be given in mental health counseling for adolescents | National scope | <https://www.gob.pe/institucion/minsa/informes-publicaciones/1893900-derecho-de-los-adolescentes-al-paquete-basico-de-atencion-integral-de-salud-en-el-contexto-de-la-covid-19-rotafolio-dirigido-al-personal-de-salud-que-atiende-a-la-poblacion-adolescente> |
| 38 | **Documento Técnico: "Lineamientos para la Asistencia Técnica y supervisión del Ministerio de Salud a los Gobiernos Regionales para el fortalecimiento de la respuesta frente a la pandemia para la COVID-19"** | MoH/ General Directorate of Disaster Risk Management and National Defense in Health | 3/18/2021 | Supervision of the implementation of mental health services through telemedicine and virtual applications for health care | All MoH organs and Regional Governments | <https://cdn.www.gob.pe/uploads/document/file/1743219/Documento%20t%C3%A9cnico%20para%20la%20Asistencia%20T%C3%A9cnica%20y%20supervisi%C3%B3n%20del%20Ministerio%20de%20Salud%20a%20los%20Gobiernos%20Regionales.pdf> |
| 39 | **Recomendaciones clínicas para la atención domiciliaria de pacientes con Covid-19** | EsSalud/ IETSI | 4/1/2021 | Provide recommendations on anxiety management for patients suspected of or confirmation of COVID-19. | All health centers of Essalud | <http://www.essalud.gob.pe/ietsi/pdfs/guias/reportes/RECOM_CLINICAS_EN_ATENCION_DOMIC_PAC_COVID19.pdf> |
| 40 | **Recomendaciones clínicas para el manejo de pacientes con COVID-19 en los centros de atención y aislamiento temporal** | EsSalud/ IETSI | 5/1/2021 | Provide clinical recommendations for patient care and follow-up with COVID-19 in the Temporary Care and Isolation Centers | All care and temporary isolation centers | <http://www.essalud.gob.pe/ietsi/pdfs/guias/reportes/Recomendaciones_manejo_de_COVID19_en_CAAT_Mayo2021.pdf> |
| 41 | **Convenio 035-2021-MoH** | MoH/ General Office of Planning, Budget, and Modernization | 5/29/2021 | The purpose of the Management Agreement is to establish the obligations that the parties will assume for the implementation of the annual economic delivery that will be granted to health personnel for the fulfillment of institutional goals. | NIMH "Honorio Delgado-Hideyo Noguchi" | [https://cdn.www.gob.pe/uploads/document/file/1919171/Convenio%20Nº%20035-2021-MINSA.pdf?v=1622349898](https://cdn.www.gob.pe/uploads/document/file/1919171/Convenio%20N%C2%BA%20035-2021-MINSA.pdf?v=1622349898) |
| 42 | **Lineamientos clínicos de manejo domiciliario de síntomas en pacientes con enfermedad terminal** | EsSalud/ IETSI | 8/1/2021 | Evidence-based clinical guidelines for the adequate home management of terminally ill patients in palliative care | All health centers performed in the home context of Essalud. | <http://www.essalud.gob.pe/ietsi/pdfs/guias/Lineamientos_cuidados_paliativos_Dolor_delirium_constipacio_06Ago2021.pdf> |
| 43 | **Recomendaciones clínicas para la evaluación y seguimiento remoto durante pandemia de SARS-COV-2 (COVID-19)** | EsSalud/ IETSI | 12/2021 | Evidence-based clinical recommendations for the remote assessment and follow-up during the COVID-19 pandemic | All health centers from Essalud. | <https://ietsi.essalud.gob.pe/wp-content/uploads/2021/12/Recomendaciones-manejo-remoto-covid-19-Actualizacion-Diciembre.pdf> |
| 44 | **Plan de Estrategia Publicitaria 2022 del Ministerio de Salud** | MoH / General Office of Communications | 2/23/2022 | Approval of the Advertising Strategy Plan of the Ministry of Health | National scope | <https://www.gob.pe/institucion/minsa/normas-legales/2768096-092-2022-minsa> |
| 45 | **Plan de Comunicación Interna 2022** | MoH/ General Office of Human Resources Management | 3/29/2022 | Approve the 2022 Internal Communication Plan of the Central Administration of the Ministry of Health | All MoH organs | [https://cdn.www.gob.pe/uploads/document/file/2965630/Resolución%20Directoral%20N°%20175-2022-OGGRH-MINSA.pdf?v=1648574842](https://cdn.www.gob.pe/uploads/document/file/2965630/Resoluci%C3%B3n%20Directoral%20N%C2%B0%20175-2022-OGGRH-MINSA.pdf?v=1648574842) |
| 46 | **Plan Operativo Institucional Anual 2021** | MoH / General Office of Legal Advice | 4/28/2022 | Approve the Modified Annual Institutional Operational Plan 2021 V.02 of Document 011: Ministry of Health | All MoH organs | <https://cdn.www.gob.pe/uploads/document/file/3048928/Plan%20Operativo%20Institucional%202021%20V.02.pdf?v=1651178912> |
| 47 | **Decreto de Urgencia que establece diversas medidas excepcionales y temporales para garantizar la atención integral en salud en respuesta a la emergencia sanitaria por la pandemia por la COVID-19** | President of the Council of Ministers, the Minister of Economy and Finance and the Minister of Health | 5/7/2022 | Emergency Decree establishing various exceptional and temporary measures to guarantee comprehensive health care in response to the health emergency caused by the COVID-19 pandemic. | Implementing units of the Ministry of Health, the National Institute of Health, the National Institute of Neoplastic Diseases and the health implementing units of the Regional Governments. | <https://www.gob.pe/institucion/minsa/normas-legales/2951190-009-2022> |
| 48 | **Modificar la Directiva Administrativa N° 287-MINSA/2020/DGIESP, Directiva Administrativa que regula los procesos, registros y accesos a la información para garantizar el seguimiento integral de los casos sospechosos y confirmados de COVID-19 (Sistema Integrado para COVID-19 – SISCOVID-19)** | MoH/ General Directorate of Strategic Interventions in Public Health | 5/8/2022 | Modify the Administrative Directive No. 287-MINSA/2020/DGIESP that regulates the processes, records and access to information to guarantee the comprehensive monitoring of suspected and confirmed cases of COVID-19 (Integrated System for COVID-19 – SISCOVID-19) | National scope | <https://www.gob.pe/institucion/minsa/normas-legales/563115-263-2020-minsa> |
| 49 | **Resolución Ministerial No. 285-2020-MINSA** | MoH / General Office for International Technical Cooperation | 5/14/2022 | Accept the donation made by the foreign institution Partners In Health in favor of the Ministry of Health, with final destination of the National Institute of Health | National scope | <https://cdn.www.gob.pe/uploads/document/file/710155/RM_285-2020-MINSA.PDF?v=1589473167> |
| 50 | **Plan Operacional Institucional Multianual (POI) 2023-2025** | MoH/ General Office of Planning, Budget, and Modernization | 5/21/2022 | Approve the Multiannual Institutional Operational Plan (POI) 2023-2025 | National scope | <https://www.gob.pe/institucion/minsa/normas-legales/3002476-367-2022-minsa> |
| 51 | **Plan estratégico multisectorial al 2030 de la política nacional multisectorial de salud "Perú, país saludable"** | MoH | 6/22/2022 | National Multisectoral Health Policy to promote healthy habits and lifestyles, ensuring comprehensive health services, and improving the population's quality of life. | National scope | [https://cdn.www.gob.pe/uploads/document/file/3287193/Plan%20estratégico%20multisectorial%20al%202030%20de%20la%20política%20nacional%20multisectorial%20de%20salud%20"Perú%2C%20país%20saludable".pdf?v=1655927840](https://cdn.www.gob.pe/uploads/document/file/3287193/Plan%20estrat%C3%A9gico%20multisectorial%20al%202030%20de%20la%20pol%C3%ADtica%20nacional%20multisectorial%20de%20salud%20%22Per%C3%BA%2C%20pa%C3%ADs%20saludable%22.pdf?v=1655927840) |
| 52 | **Resolución Ministerial N.° 490-2022-MINSA** | MoH/ General Office of Planning, Budget, and Modernization | 7/5/2022 | Allocation of Resources to Finance the Extension of the Contract for COVID-19 Personnel | National scope | [https://cdn.www.gob.pe/uploads/document/file/3354197/Resolución%20Ministerial%20N°%20490-2022-MINSA.pdf?v=1657054988](https://cdn.www.gob.pe/uploads/document/file/3354197/Resoluci%C3%B3n%20Ministerial%20N%C2%B0%20490-2022-MINSA.pdf?v=1657054988) |
| 53 | **Resolución Ministerial N.° 558-2022-MINSA** | MoH/ General Office of Planning, Budget, and Modernization | 7/26/2022 | Authorize the incorporation of increased public revenues in the Institutional Budget the Ministry of Health for 2022 | National scope | [https://cdn.www.gob.pe/uploads/document/file/3453131/Resolución%20Ministerial%20Nº%20558-2022-MINSA.pdf?v=1658925262](https://cdn.www.gob.pe/uploads/document/file/3453131/Resoluci%C3%B3n%20Ministerial%20N%C2%BA%20558-2022-MINSA.pdf?v=1658925262) |
| 54 | **Prioridades Nacionales de Investigación en COVID-19 (SARS-CoV2) y otros virus respiratorios con potencial pandémico: Preparándonos para la siguiente pandemia, 2022-2026** | MoH / General Office of Legal Advice | 9/15/2022 | Approve the "National Research Priorities for COVID-19 (SARS-CoV-2) and Other Respiratory Viruses with Pandemic Potential: Preparing for the Next Pandemic, 2022-2026" | National scope | [https://cdn.www.gob.pe/uploads/document/file/3642092/Resolución%20Ministerial%20%20N°%20711-2022-MINSA.pdf?v=1663277741](https://cdn.www.gob.pe/uploads/document/file/3642092/Resoluci%C3%B3n%20Ministerial%20%20N%C2%B0%20711-2022-MINSA.pdf?v=1663277741) |
| 55 | **Resolución Ministerial N.° 850-2022-MINSA** | MoH /General Directorate of Strategic Interventions in Public Health | 10/20/2022 | Approve the "Technical guide for the rehabilitation of people affected by COVID-19" | National scope | <https://www.gob.pe/institucion/minsa/normas-legales/3614919-850-2022-minsa> |
| 56 | **Resolución Ministerial N.° 1124-2022-MINSA** | MoH/ General Office of Planning, Budget, and | 1/2/2023 | Authorize the incorporation of increased public revenues in the Institutional Budget the Ministry of Health for 2023 | National scope | <https://www.gob.pe/institucion/minsa/normas-legales/3817781-1124-2022-minsa> |
| 57 | **Resolución Ministerial N.° 137-2023-MINSA** | MoH /General Directorate of Strategic Interventions in Public Health | 2/10/2023 | Approve the technical health standard "Management of adults with post-COVID-19 (prolonged COVID)". | National scope | <https://www.gob.pe/institucion/minsa/normas-legales/3882032-137-2023-minsa> |

Note: CMHC = Community Mental Health Centers; MoH = Ministry of Health
