# Supplementary material for: Scoping review of mental health-related policies issued in the context of the COVID-19 pandemic in Peru
Source: PLOS Ment Health. 2026 Apr 27;3(4):e0000459. doi: 10.1371/journal.pmen.0000459 (PMC13120698; doi:10.1371/journal.pmen.0000459)
Supplement: S6 File — (DOCX) [file pmen.0000459.s006.docx]

**Supporting information 6. Key definitions**

| **Key definitions** |
| --- |
| - **Community Mental Health Centers (CMHCs).** Offer specialized outpatient care to patients with serious or complex mental disorders or psychosocial problems. Each CMHC has a multidisciplinary team, including psychiatrists, psychologists, nurses, nursing technicians, social workers, medical technologists, pharmaceutical chemists and speech therapists**.** - **First psychological aids**. Are part of the psychosocial support for care of the mental health of people who are going through a crisis situation. Its objective is to facilitate the activation of a person or group, strategies of effective coping in the face of crisis, it is say, develop or strengthen those ways of thinking and acting that facilitate the resolution of a situation perceived as demanding. - **Follow-up.** Activities aimed at knowing the clinical evolution of the patient and early identification of warning signs. - **Personal protective equipment (PPE).** Devices, materials and personal clothing intended for each worker to protect him from one or more risks present at work and that may threaten his safety and health. PPEs are a temporary and complementary alternative to measures collective prevention. It consists of: apron, N95 respirator or higher, surgical mask, protective glasses, face shield, shoe protector, work shoe and hat; according to occupational risk. - **Psychosocial support.** Set of activities developed by health personnel to protect, promote autonomy and participation of people affected by a crisis situation considering your resources and rights. - **Psychosocial support teams.** Responsible for monitoring occupational psychosocial risks, implementation and monitoring of self-care measures, mental health care, identification of mental health problems, psychosocial support, management for intervention and monitoring for the recovery of patients with COVID-19 and health personnel. - **Self-care.** Capacity of individuals, families and communities to promote health, prevent disease, maintain health and cope with illnesses and disabilities with or without the support of health personnel. - **Women's Emergency Center (WEC).** The WECs are specialized and free public services for care and prevention of family and sexual violence, created by the Ministry of Women and Vulnerable Populations. WECs provide legal information, social orientation, defense, judicial and psychological support. |
